# Association of delayed chemoradiotherapy with elevated Epstein-Barr virus DNA load and adverse clinical outcome in nasopharyngeal carcinoma treatment during the COVID-19 pandemic: a retrospective study

**DOI:** 10.1186/s12935-022-02748-y

**Published:** 2022-10-31

**Authors:** Cheng-Long Huang, Xue-Liang Fang, Yan-Ping Mao, Rui Guo, Wen-Fei Li, Si-Si Xu, Jun Ma, Lei Chen, Ling-Long Tang

**Affiliations:** 1 Department of Radiation Oncology, State Key Laboratory of Oncology in South China, Collaborative Innovation Center for Cancer Medicine, Guangdong Key Laboratory of Nasopharyngeal Carcinoma Diagnosis and Therapy, Sun Yat-sen University Cancer Center, 651 Dongfeng Road East, 510060 Guangzhou, China; 2 Department of Otolaryngology, Peking Union Medical College Hospital, Research Units of New Technologies of Endoscopic Surgery in Skull Base Tumor, Chinese Academy of Medical Sciences and Peking Union Medical College, Beijing, China; 3 National Cancer Center/National Clinical Research Center for Cancer/Cancer Hospital & Shenzhen Hospital, Chinese Academy of Medical Sciences and Peking Union Medical College, Shenzhen, China

**Keywords:** Treatment delay, EBV DNA, Nasopharyngeal carcinoma, Clinical outcomes, COVID-19 pandemic

## Abstract

**Background:**

To summarize the impact of radiotherapy (RT) and chemotherapy delays on patients with nasopharyngeal carcinoma (NPC) during the COVID-19 pandemic.

**Methods:**

We retrospectively included 233 patients with stage II-IVa NPC treated with RT and chemotherapy between December 11, 2019 and March 11, 2020. The outcomes were elevation in the EBV DNA load between two adjacent cycles of chemotherapy or during RT, and 1-year disease-free survival (DFS).

**Results:**

RT delay occurred in 117 (50%) patients, and chemotherapy delay occurred in 220 (94%) patients. RT delay of ≥ 6 days was associated with a higher EBV DNA elevation rate (20.4% vs. 3.6%, odds ratio [OR] = 6.93 [95% CI = 2.49–19.32], P < 0.001), and worse 1-year DFS (91.2% vs. 97.8%, HR = 3.61 [95% CI = 1.37–9.50], P = 0.006), compared with on-schedule RT or delay of < 6 days. Chemotherapy delay of ≥ 10 days was not associated with a higher EBV DNA elevation rate (12.5% vs. 6.8%, OR = 1.94 [95% CI = 0.70–5.40], P = 0.20), or worse 1-year DFS (93.8% vs. 97.1%, HR = 3.73 [95% CI = 0.86–16.14], P = 0.059), compared with delay of < 10 days. Multivariable analyses showed RT delay of ≥ 6 days remained an independent adverse factor for both EBV DNA elevation and DFS.

**Conclusion:**

To ensure treatment efficacy for patients with nonmetastatic NPC, initiation of RT should not be delayed by more than 6 days; the effect of chemotherapy delay requires further investigation.

**Supplementary Information:**

The online version contains supplementary material available at 10.1186/s12935-022-02748-y.

## Background

The COVID-19 pandemic has had a serious impact on health care services all over the world. Cancer patients, in particular, face the risk of not receiving timely and adequate oncologic care. The reasons for this are restrictions on the movement of people as a result of nationwide lockdown implemented in many countries, the limited health care resources as a result of diversion of resources to tackling the pandemic, and patient’s fear of COVID that makes them avoid clinics/hospitals. For example, the Philippine General Hospital Cancer Institute discontinued operations for one week, since the medical oncologists were sent to the frontline of the COVID-19 response [1]. Further, at Zhongnan Hospital in Wuhan, China, the mean number of radiotherapy caseloads per day decreased sharply from 188 to 12 during the COVID-19 outbreak [2]. Another concern is the increased risk of COVID-19 infection as a result of nosocomial transmission and compromised immunity caused by the cancer itself and/or cancer treatment [3, 4]. Despite these concerns, the risk associated with delay of treatment is unclear and has attracted widespread attention from experts, and several guidelines relevant to this have been published [5, 6]. According to early published data from China, in the population infected with SARS-CoV-2, the risk of cancer patients developing severe events is nearly five times higher than that in non-cancer patients [7]. Further, it has been suggested that chemotherapy be postponed in endemic areas [7]. Given these findings, it is important to assess the risks of administering cancer treatment or delaying it in the context of the ongoing pandemic.

Nasopharyngeal carcinoma (NPC) is one of the most common head and neck cancers in southern China. According to the NCCN guidelines, induction chemotherapy (IC) combined with concurrent chemoradiotherapy (CCRT) is recommended for locally advanced, nonmetastatic (stage II–IVa) NPC [8]. For head and neck cancer care in the time of COVID-19, some experts recommend that RT be initiated immediately [5], while some recommend that initiation of RT should not be delayed by more than 4–6 weeks [6]. Since NPC is highly prevalent in China, it is important to investigate the risks associated with treatment delay. Therefore, in the present study, we have aimed to shed light on the potential implications of the SARS-CoV-2 crisis for cancer management. To this end, we retrospectively reviewed cases of nonmetastatic NPC in which the IC and/or CCRT plan was delayed during the pandemic. Changes in the Epstein-Barr virus (EBV) DNA load of patients were measured, as plasma EBV DNA load is one of the most important biomarkers for risk stratification and disease surveillance during and after treatment, and residual EBV DNA load indicates a poor prognosis [9–12]. 1-year disease-free survival (DFS) was also calculated to summarize the effect of treatment delay.

## Methods

### Patients

This retrospective review included patients who received intensity-modulated radiotherapy (IMRT) between December 11, 2019, and March 11, 2020, at our cancer center. The inclusion criteria were (1) histologic confirmation of nonkeratinizing NPC and (2) newly diagnosed stage II–IVa disease according to the 8th edition of the American Joint Committee on Cancer/Union for International Cancer Control (AJCC/UICC) staging system. The exclusion criteria were (1) treatment with palliative intent and (2) recurrence and/or metastasis. Based on these criteria, a total of 233 patients were selected for this study. All the patients had undergone a comprehensive pre-treatment evaluation, including physical examination, nasopharyngeal and neck magnetic resonance imaging, chest radiography, abdominal sonography, and whole-body bone scan or (18)F-fluorodeoxyglucose positron emission tomography/computerized tomography examination. Plasma EBV DNA measurement was performed as described previously [12]. A pretreatment EBV DNA cutoff value of 2000 copies/ml was adopted [13].

The ethical review board of Sun Yat-sen University Cancer Centre approved of this retrospective analysis of anonymized patient data, and waived the need for written consent from the included patients. However, the oral consent of the patients was obtained via telephone and documented by telephone recording.

### Institutional guidelines for treatment

Our institutional guidelines recommend radiotherapy (RT) combined with concurrent chemotherapy (CRT) and/or IC for stage II–IVa NPC. Three regimens of IC are frequently used: gemcitabine (1 g/m^2^ on days 1 and 8) plus cisplatin (80 mg/m^2^ on day 1), cisplatin (75 mg/m^2^) with docetaxel (75 mg/m^2^), and cisplatin (60 mg/m^2^) plus docetaxel (60 mg/m^2^) with 5-fluorouracil (600–750 mg/m^2^ per day for 5 days), triweekly, for 3 cycles. In cases where IC is also administered, CCRT is usually initiated 3 weeks after the last cycle of IC. CRT usually consists of cisplatin (80–100 mg/m^2^) triweekly for 3 cycles. Adjuvant chemotherapy is a less frequently used option due to poor compliance. All patients were treated with IMRT. Target volumes were delineated according to an individualized delineation protocol [14]. The prescribed doses were according to those reported previously [13]. All targets were treated simultaneously using the simultaneous integrated boost technique.

Chemotherapy was considered to be delayed if the interval between two adjacent cycles was longer than the recommended interval, which was usually 21 days.

### Endpoint and follow up

The study endpoints were elevated EBV DNA load (with a cutoff of 5% above the previous value to account for baseline fluctuation or variation) between two adjacent cycles of chemotherapy or during RT, and DFS (defined as the time from the initiation of treatment to documented disease relapse [locoregional relapse or distant metastasis] or death from any cause, whichever occurred first).

Patients were followed up at least every 3 months during the first 2 years. The median follow-up duration was 17.7 months (range, 4.0–22.2 months; interquartile range, 16.0–19.6 months).

### Statistical analysis

Receiver operator characteristic (ROC) analysis was used to calculate the cut-off value for RT and chemotherapy delay that would be predictive of elevation in the EBV DNA load. Logistic regression model was performed to calculate the odds ratio (OR) and their associated 95% confidence intervals (CIs), and to perform multivariable analyses to identify significant independent factors for the elevation in the EBV DNA load. Kaplan–Meier curves were used to present time-to-event data, and difference was assessed using log-rank tests. Cox proportional hazard model was used to calculate the hazard ratios (HR), and to perform multivariable analyses to identify significant independent factors for DFS. All analyses were performed using SPSS version 25.0 (IBM Corporation, Armonk, NY, USA). Two-tailed *P*-values < 0.05 were considered to indicate statistical significance.

## Results

### Patient characteristics and treatment

Table 1 depicts the baseline characteristics and treatment modalities used in the 233 patients. The majority of the patients (97%) had stage III–IVa disease; 89% had T3-4 disease; and 65% had N2–3 disease. In 161 (69%) patients, the pretreatment EBV DNA load was < 2000 copies/ml, while the remaining 72 (31%) patients had a pretreatment EBV DNA load of ≥ 2000 copies/ml.

**Table 1:** Clinical characteristics of the included 233 patients

Characteristic	No. (%)
**Sex**	
Male	161 (69%)
Female	72 (31%)
**Age (years)**	
≤ 46	119 (51%)
> 46	114 (49%)
**Pretreatment EBV DNA**	
≤ 2000 copies/ml	161 (69%)
> 2000 copies/ml	72 (31%)
T stage*	
T1	4 (2%)
T2	23 (10%)
T3	144 (62%)
T4	62 (27%)
N stage*	
N0	14 (6%)
N1	68 (29%)
N2	86 (37%)
N3	65 (28%)
Overall stage*	
II	8 (3%)
III	114 (49%)
IVa	111 (48%)
**Induction chemotherapy**	
No	37 (16%)
Yes	196 (84%)
**Concurrent chemotherapy**	
No	5 (2%)
Yes	228 (98%)
**Chemotherapy delay**	
On schedule or delay < 10 days	70 (30%)
With delay ≥ 10 days	163 (70%)
**Radiotherapy**	
On schedule or delay < 6 days	140 (60%)
With delay ≥ 6 days	93 (40%)

Percentages may not total 100 because of rounding

*According to the 8th edition of the AJCC staging system

All the included patients had completed RT for at least 30 fractions (range, 30–35 fractions) with or without IC and/or CRT prior to the start of this study. 196 (84%) patients received IC: 12 had undergone 1 cycle, and 184 had undergone 2–4 cycles. Further, 228 (98%) patients had undergone CRT: 14 had received 1 cycle, and 214 had received 2–4 cycles. Only 13 (6%) of the 233 patients received all the cycles of chemotherapy on schedule, while in 220 (94%) patients, chemotherapy was delayed (median, 16 days; range, 1–56 days; interquartile range [IQR], 9–21 days). Further, in 116 (50%) patients, RT was initiated on schedule, while in 117 (50%) patients, RT was delayed (median, 13 days; range, 1–39 days; IQR, 7–20 days).

### Association of treatment delay with elevation in the EBV DNA load

The EBV DNA load was found to have elevated between IC1 and IC2 (n = 1), post-IC and pre-CCRT (n = 22), and between CRT1 and CRT2 (n = 2). ROC analysis revealed that the optimal cutoff value for RT and chemotherapy delay that was predictive of elevation in the EBV DNA load was 6 and 10 days, respectively (Figure S1 and Table S1).

**Fig. 1:**
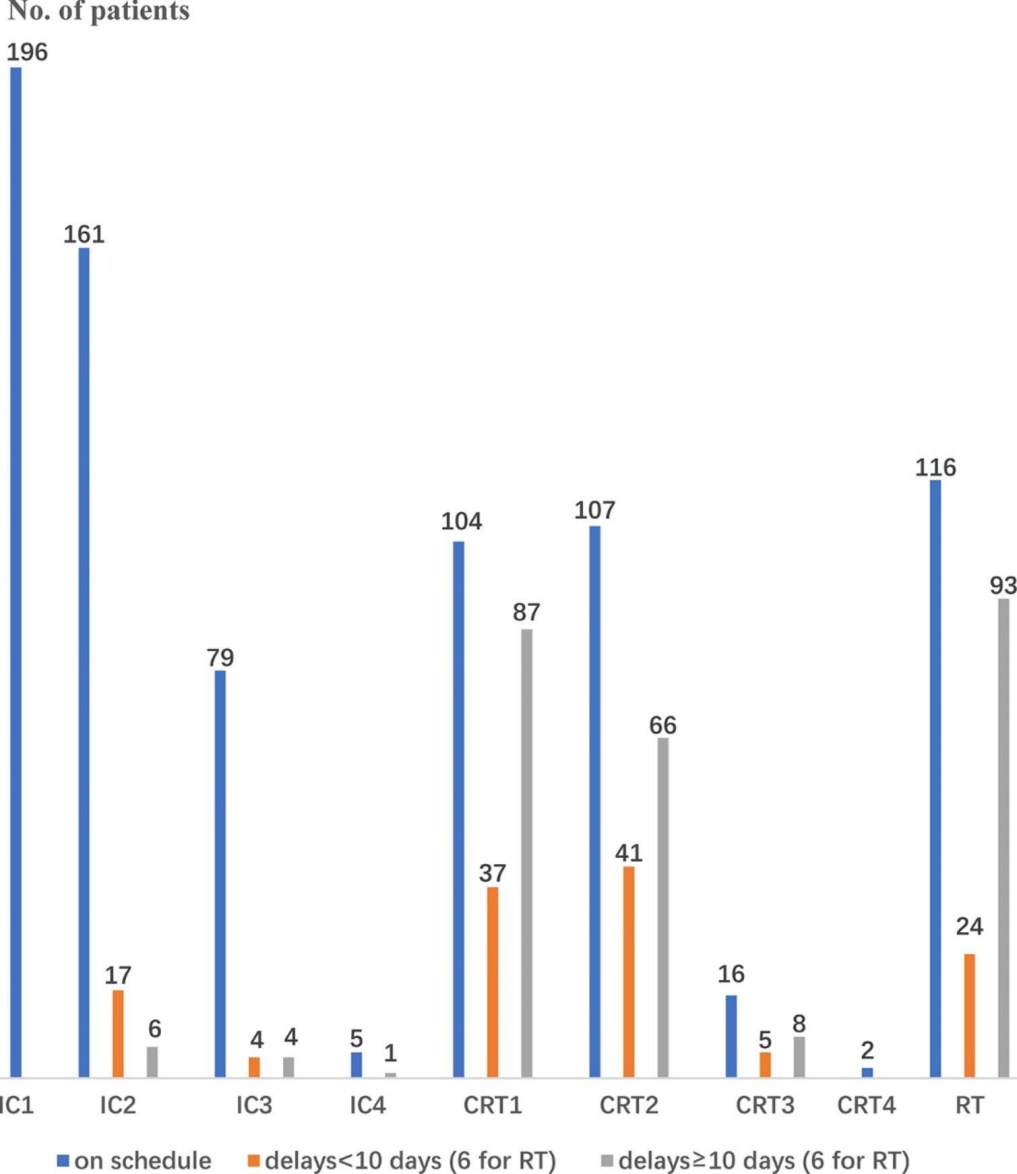
Delay conditions about every cycle of chemotherapy and radiotherapy. IC: induction chemotherapy; CRT: concurrent chemotherapy; RT: radiotherapy

Figure 1 presents detailed information about delays for every cycle of chemotherapy and RT. In short, 11 (6%) patients’ IC plan and 153 (67%) patients’ CRT plan were delayed by ≥ 10 days (median = 18, range, 10–56; IQR, 15–25 days). Further, 93 (40%) patients’ RT plan was delayed by ≥ 6 days (median = 16, range, 6–39; IQR, 11–22 days). Patients with RT delay of ≥ 6 days were more likely to have a subsequently elevated EBV DNA load than those for whom the RT plan was initiated on schedule or delayed by < 6 days (20.4% vs. 3.6%, odds ratio [OR] = 6.93 [95% CI = 2.49–19.32], *P* < 0.001). Chemotherapy delay of ≥ 10 days was not associated with elevated EBV DNA, as compared with a delay of < 10 days (12.5% vs. 6.8%, OR = 1.94 [95% CI = 0.70–5.40], *P* = 0.20). When adjusted for other clinical factors in multivariable analysis, RT delay of ≥ 6 days remained an adverse factor for a subsequently elevated EBV DNA load (OR = 7.08 [95% CI = 2.53–19.88], P < 0.001) (Table 2).

**Table 2:** Summary of multivariable analysis of prognostic factors for EBV DNA elevation and DFS

Endpoint	Variable	HR	95% CI	*P* value*
EBV DNA elevation	RT delay (≥ 6 vs. < 6 days)	7.08	2.53–19.88	**< 0.001**
	Pre-DNA (≥ 2000 vs. <2000 copies/ml)	2.17	0.88–5.32	0.092
DFS	RT delay (≥ 6 vs. < 6 days)	3.61	1.37–9.50	**0.009**

Abbreviations: RT, radiotherapy; Pre-DNA, pretreatment EBV DNA; DFS, disease-free survival; HR, hazard ratio; CI, confidence interval

* P-values were calculated using an adjusted Cox proportional hazards model; the following concerned prognostic variables were included in the analysis of EBV DNA elevation: age (≥ 45 vs. <45 years), sex (female vs. male), T category (T3-4 vs. T1-2), N category (N2-3 vs. N0-1), pretreatment EBV DNA (≥ 2000 vs. <2000 copies/ml), induction chemotherapy (yes vs. no), and RT delay (≥ 6 vs. < 6 days). In analysis of DFS, chemotherapy delay (≥ vs. < 10 days) was also included. Insignificant factors were excluded from the model and were not shown in the results

Since most of the EBV DNA load (22/25) elevated between IC and CCRT, we used post-IC EBV DNA as a hierarchical factor and reanalyzed the association between treatment delay and changes in the EBV DNA load. We found that patients with an RT delay of ≥ 6 days were at a higher risk of elevated EBV DNA load than those with an RT delay of < 6 days, regardless of whether EBV DNA after IC was detectable (elevated rate: 52·4% vs. 21·1%, OR = 4·13 [1.02–16.67], *P* = 0·041) or not (12·9% vs. 1·3%, OR = 11·56 [1.40-95.09], *P* = 0·011).

### Association of treatment delay with DFS

The median follow-up time for DFS was 17.7 months (IQR, 16.0-19.7). During the follow-up, 9 patients had loco/regional relapse, 11 had distant metastasis, and 2 died.

**Fig. 2:**
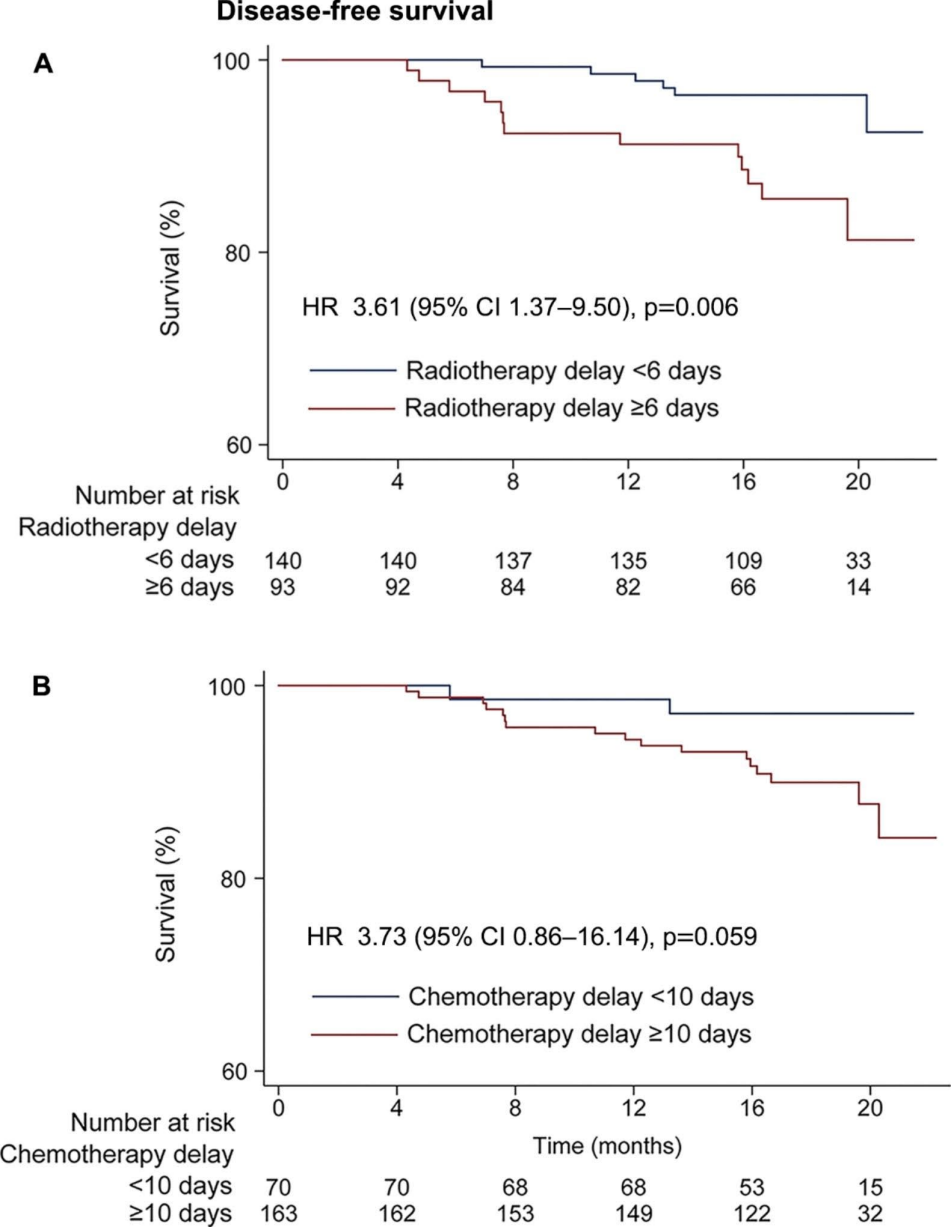
Kaplan–Meier curves showing the disease-free survival according to patients with and without radiotherapy delay of ≥ 6 days (A) and patients with and without chemotherapy delay of ≥ 10 days (B)

Univariate analysis showed RT delay of ≥ 6 days was associated with worse 1-year DFS (91.2% vs. 97.8%, HR = 3.61 [95% CI = 1.37–9.50], P = 0.006), compared with on-schedule RT or delay of < 6 days; chemotherapy delay of ≥ 10 days was associated with non-significant worse 1-year DFS (93.8% vs. 97.1%, HR = 3.73 [95% CI = 0.86–16.14], P = 0.059), compared with delay of < 10 days (Fig. 2). In multivariable analysis adjusted for other clinical factors, RT delay of ≥ 6 days remained an independent adverse factor for DFS (HR = 3.61 [1.37–9.50], P = 0.009), while chemotherapy delay of 10 days was not (Table 2).

### Association of EBV DNA elevation during treatment with DFS

**Fig. 3:**
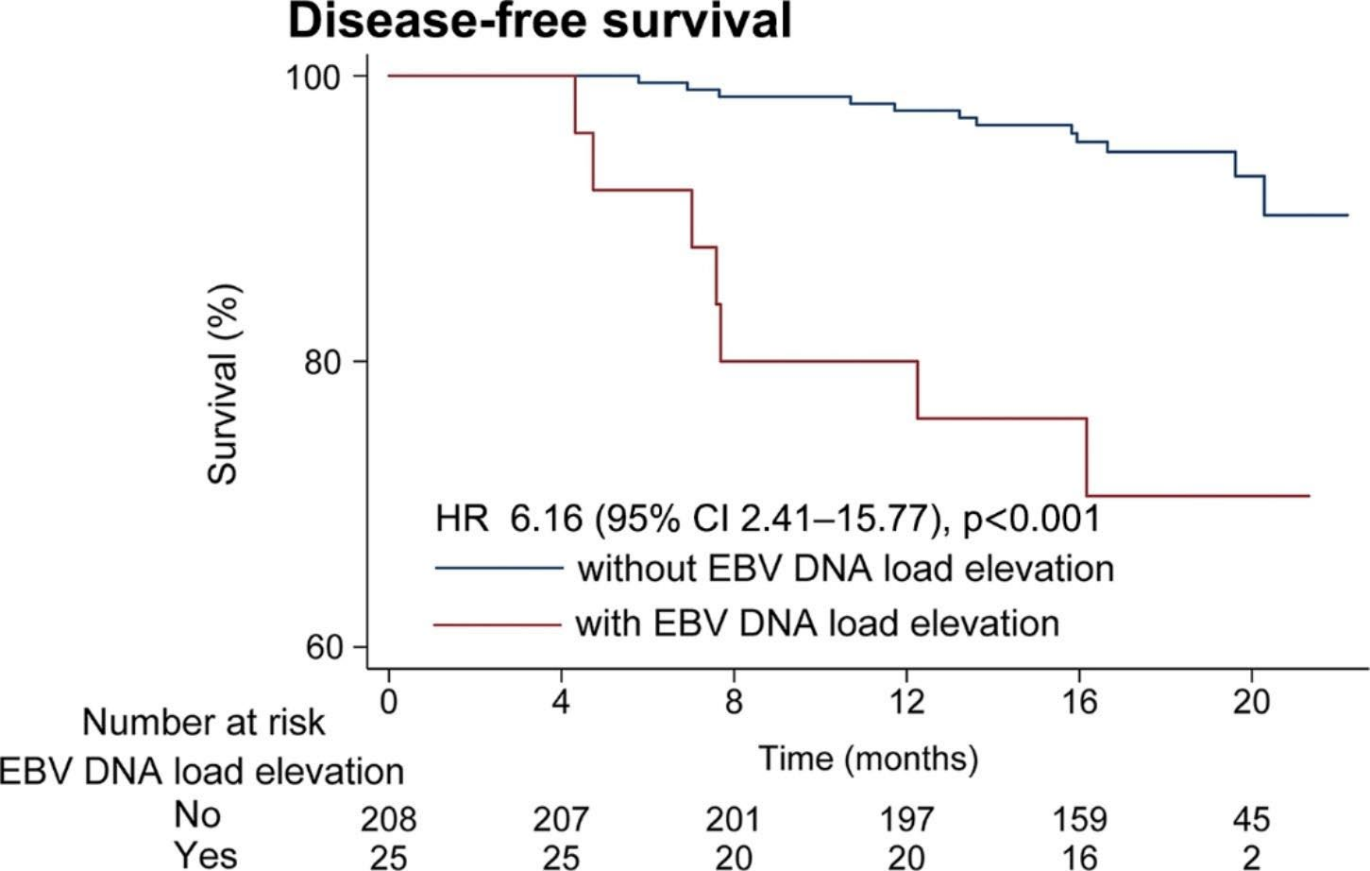
Kaplan–Meier curves showing the disease-free survival according to patients with and without EBV DNA load elevation during treatment

Patients with EBV DNA elevation during treatment had a worse 1-year DFS (76.0% vs. 97.6%, HR = 6.16 [2.41, 15,77], P < 0.001), compared with those without EBV DNA elevation (Fig. 3). In multivariable analysis, EBV DNA elevation during treatment remained an independent adverse factor for DFS (HR = 6.16 [2.41-15,77], P < 0.001).

## Discussion

In the present study, we respectively reviewed 233 NPC patients who had received treatment at our cancer center during the COVID-19 pandemic in China, and described their treatment and short-term outcomes. We found that when the RT plan was delayed by ≥ 6 days, the EBV DNA load, one of the most important prognostic factors for NPC, was more likely to be subsequently elevated; further, patients with a RT delay of ≥ 6 days had worse 1-year DFS.

Plasmas EBV DNA load is an indicator of tumor load and is widely used for risk stratification and disease surveillance before and after treatment [10, 11]. It has been reported that detectable EBV DNA after completion of IC, at the midpoint of CCRT, and after RT are all indicative of poor survival outcomes [9, 11, 12]. Moreover, elevation in EBV DNA during IC or CCRT is associated with poor DFS [15], and increase in EBV DNA during the follow-up can correctly predict recurrences [16] and metastatic failure [17]. Therefore, EBV DNA is a useful biomarker of disease status and survival outcomes, and this is why we chose it as one of the study endpoints.

We found that the EBV DNA load was more likely to be elevated subsequently when RT was delayed for over a certain period of time. The sudden increase in tumor load might be a result of accelerated repopulation of the surviving tumor clonogens [18, 19]. Withers et al. [19] found a daily decrease of over 1% in the tumor control rate if RT was interrupted and prolonged, and they attributed this to accelerated repopulation. Therefore, as the tumor load increased, the concentration of EBV DNA released into the plasma increased. Considering that there might exist a latent time within which the accelerated repopulation occurs, we performed ROC analysis to determine the optimal cut-off point for treatment delay, and determined that it was ≥ 6 days for RT and ≥ 10 days for chemotherapy. Similar cutoff values for RT delay have been described for long-term prognosis in previous studies, which have indicated that a prolonged interval of > 30 days [20] (i.e., delay of > 9 days) between IC and RT was associated with worse 5-year overall survival and DFS. In the present study, we also found that patients with a RT delay of ≥ 6 days had worse 1-year DFS, and RT delay of ≥ 6 days remained an independent adverse factor for DFS when adjusted for other factors. Although the follow-up time is relatively short, the adverse effect of RT delay on prognosis has been shown.

In the present study, 39.9% (93/233) of patients treated in our hospital had a RT delay of ≥ 6 days during the pandemic, which was much higher than that before the pandemic (≥ 7 days: 10.3% [807/7826])[21]. The COVID-19 pandemic did seriously affect cancer treatment. Based on our results, we recommend that the treatment of patients with nonmetastatic NPC should not be delayed for too long, in order to ensure good long-term survival, despite the limitations posed by the COVID-19 pandemic. It is critical for both physicians and patients to weigh the risk of adverse effects that could result from delay of cancer treatment against the risk of exposure to COVID-19 infection [5]. To this end, maintaining open, online routes of communication about the condition of the disease and treatment strategy between doctors and patients is important.

## Conclusion

To conclude, based on the findings of the present study, we recommend that despite the risk and limitations posed by the COVID-19 pandemic, in patients with nonmetastatic NPC, RT be initiated without a delay of more than 6 days, to ensure long-term survival. Long-term follow-up of these patients is needed to confirm these findings.

## Electronic supplementary material

Below is the link to the electronic supplementary material.

Supplementary Figure S1 and Table S1.

## Data Availability

The datasets used and/or analysed during the current study are available from the corresponding author on reasonable request.

## Abbreviations

NPC: nasopharyngeal carcinoma
IC: induction chemotherapy
CCRT: concurrent chemoradiotherapy
RT: radiotherapy
EBV DNA: Epstein-Barr virus DNA
IMRT: intensity modulated radiotherapy
AJCC/UICC: American Joint Committee on Cancer/Union for International Cancer Control
CRT: concurrent chemotherapy
ROC: receiver operator characteristic
