# Supplementary material for: Association of delayed chemoradiotherapy with elevated Epstein-Barr virus DNA load and adverse clinical outcome in nasopharyngeal carcinoma treatment during the COVID-19 pandemic: a retrospective study
Source: Cancer Cell Int. 2022 Oct 31;22:331. doi: 10.1186/s12935-022-02748-y (PMC9623943; doi:10.1186/s12935-022-02748-y)
Supplement: Supplementary file 1 — Supplementary Figure S1 and Table S1. [file 12935_2022_2748_MOESM1_ESM.docx]

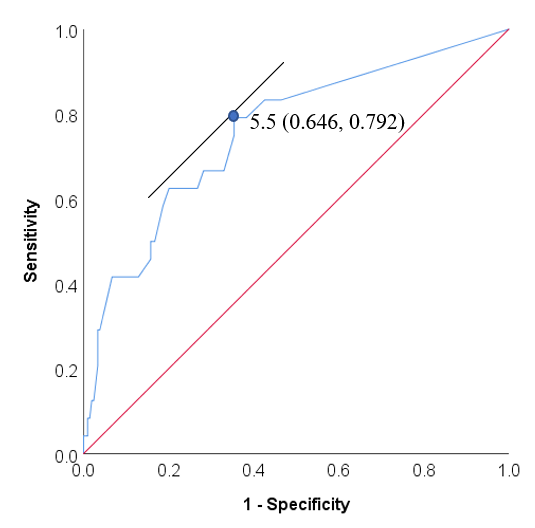


Figure S1. Receiver operating curve analysis evaluating the predictive value of radiotherapy delay for elevation in the EBV DNA load. The optimal cutoff value of radiotherapy delay was 5.5 days, with a specificity of 0.646 and a sensitivity of 0.792.

| Radiotherapy delay (days) | Odds ratio (95% CI) | P value |
| --- | --- | --- |
| >1 vs <1 | 5.77 (1.91, 17.47) | 0.002 |
| >2 vs <2 | 5.89 (1.95, 17.81) | 0.002 |
| >3 vs <3 | 6.24 (2.06, 18.88) | 0.001 |
| >4 vs <4 | 6.74 (2.23, 20.42) | 0.001 |
| >5 vs <5 | 6.13 (2.20, 17.06) | 0.001 |
| >6 vs <6 | **6.93** (2.49, 19.32) | <0.001 |
| >7 vs <7 | 5.47 (2.08, 14.39) | 0.001 |
| >8 vs <8 | 4.06 (1.66, 9.94) | 0.002 |
| >9 vs <9 | 4.74 (1.93, 11.65) | 0.001 |
| >10 vs <10 | 5.09 (2.07, 12.51) | <0.001 |
| >11 vs <11 | 4.55 (1.89, 10.99) | 0.001 |
| >12 vs <12 | 5.44 (2.24, 13.20) | <0.001 |
| >13 vs <13 | 5.75 (2.36, 13.96) | <0.001 |
| >14 vs <14 | 6.63 (2.71, 16.19) | <0.001 |
| >15 vs <15 | 6.10 (2.52, 14.76) | <0.001 |

Table S1. Association of radiotherapy delay with elevation in the EBV DNA load.
